# Understanding the Impact of Urinary Incontinence in Persons with Dementia: Development of an Interdisciplinary Service Model

**DOI:** 10.1155/2021/9988056

**Published:** 2021-06-19

**Authors:** Patrick Juliebø-Jones, Elizabeth Coulthard, Elizabeth Mallam, Hilary Archer, Marcus J. Drake

**Affiliations:** ^1^Bristol Brain Centre, Southmead Hospital, Bristol, UK; ^2^Bristol Urological Institute, Southmead Hospital, Bristol, UK; ^3^University of Bristol, Bristol, UK

## Abstract

**Introduction:**

Prevalence of urinary symptoms such as incontinence (UI) in patients with dementia is estimated to exceed 50%. The resultant psychological and socio-economic burden can be substantial. Our aim was to develop a dedicated urology service within a cognitive impairment clinic in order to treat and better understand the bothersome urinary symptoms suffered by persons with dementia.

**Methods:**

Patients attending this clinic were invited to be assessed and interviewed by urologist, together with their family and/or carer. In addition, formal history, examination and relevant investigations, themes of importance such as quality of life, and select question items were drawn from validated questionnaires. Multidisciplinary team (MDT) meeting was carried out on the same day. Outcomes of the first 75 patients with UI and dementia have been reported.

**Results:**

Average age was 70 years (range 58–98). Majority of persons had a diagnosis of Alzheimer's disease (*n* = 43, 57%). Average score for how much urine leakage interferes with everyday life was 7.7/10 (range 2–10). 58.7% (*n* = 44) revealed some degree of sleep disturbance due to UI. 83% (*n* = 62) stated daily activities were limited due to UI. Two-thirds of persons with dementia (*n* = 50) stated their bladder problem makes them feel anxious. 88% (*n* = 67) felt the topic was socially embarrassing. All carers stated that the person's continence issues affect the care they provide. Less than one-third of carers (30.7%, *n* = 23) were aware of or had been in contact with any bladder and bowel community service. More than half of the carers (*n* = 46, 65%) were concerned incontinence may be a principal reason for future nursing home admission.

**Conclusion:**

UI can be distressing for persons with dementia. Care partners were concerned about loss of independence and early nursing home admission. Awareness of bladder and bowel services should be increased.

## 1. Introduction

The psychological and socio-economic impact of urinary incontinence (UI) can be very burdensome for patients, families, and carers. These effects are magnified when dementia is also present. Dementia will affect over 1 million people in the UK by 2021 and the prevalence of concomitant urinary symptoms such as incontinence is estimated to exceed 50% [1]. The prevalence is likely underestimated given that many are believed to not report it. Urinary incontinence (UI), defined as the complaint of involuntary loss of urine, negatively affects quality of life [2]. It is now widely recognised as a key risk factor and precipitant for premature admission to nursing home (NH) residency [3]. Independent of any physical comorbidities, dementia is also a risk factor for hospital admission [4]. However, despite the reality that bothersome urinary symptoms in this population represent a pressing research priority, as recognised by the World Health Organisation (WHO), such activity is lacking and available evidence to direct treatment pathways remains under-reported [5, 6].

Our aim was to develop and pilot a dedicated urology service within a cognitive impairment clinic led by neurologists. The principal objective of this was to improve understanding and treatment of bothersome urinary symptoms suffered by these patients, with a principal focus on patients with dementia and UI. To our knowledge, this has not been described previously. The findings of the first 75 persons with dementia and UI have been reported as well as the views of their carer.

### 1.1. Action Plan for Implementing the Intervention

The setting was a dedicated cognitive impairment outpatient clinic at the Bristol Brain Centre. This established service already implemented a multidisciplinary approach with involvement of psychologist, psychiatrist, specialist nursing team, and academic researchers in dementia.

Such as the range of complex needs in patients with dementia, there is now increasing recognition regarding the merits of such an approach in their healthcare [7]. It also allows the clinician to focus more on their area of expertise [8]. Chase et al. recently published findings from their study, which incorporated semistructured interviews, focus group discussions, and shadowing of caregivers. The conclusions revealed lack of existing integrated frameworks and the significant day-to-day challenges faced by practitioners in this regard [8].

The first step in the development of such an approach was for the urologist to adopt an observer role in all parts of this existing clinic over a three-month period. Collett et al. outlined recommendations for setting up a multidisciplinary clinic and how can it be built over time with new specialities becoming involved. The authors highlighted the importance of team members understanding the roles of their colleagues in different specialities [9]. This period of familiarisation allowed learning of the typical clinical cases attending for assessment and review as well as the organisation of the clinic.

Later, persons attending this clinic, together with their carer(s), were invited to be assessed and interviewed by a urologist. In addition, formal history, exam and relevant investigations, themes of importance such as impact on quality of life, and select question items were drawn from validated questionnaires such as the King's Health Questionnaire (KHQ) and relevant International Consultation on Incontinence Questionnaire (ICIQ) modules such as ICIQ-short form (SF) [10–14]. A round table discussion with relevant continence experts (from disciplines of urology, nursing, dementia, and elderly medicine) had taken place at start of the project to determine the themes and areas of importance. These were determined to be the interruption to activities of daily life (physical, social, and sleep), unmet care needs, stigma, and carer burden. This was led and coordinated by a research fellow in dementia. Care partners were also involved. The items generated were consistent with a scoping review carried out by Kalánková et al. and a previous European cross-sectional survey [15, 16]. This study was carried out as part of an audit of service provision and therefore no specific ethical approval was deemed to be required. The Standard for QUality Improvement Reporting Excellence (SQUIRE) checklist was adhered to [17]. Clinician funding was supplied in select cases by National Institute Health Research (NIHR).

### 1.2. Services Offered

For persons with dementia attending the clinic, the urologist routinely carried out history, physical exam, and basic tests including assessment to rule out any reversible causes of UI such as infection, constipation, restricted mobility, pharmacotherapy, and excess fluid [18]. Medication advice could be given and summarised for the persons, their carer(s), and primary care team. In persons where special tests, e.g., uroflowmetry, were required, these were referred internally. Bladder diaries are a validated tool to aid the quantitative measurement of micturition and temporal micturition patterns [19]. Persons with sufficient cognitive function were issued with such a diary to complete and return. Each person was kept on a prospective database and followed up as required. Also, recorded were the responses given to the predetermined question items drawn from the aforementioned validated questionnaires. For patients with severe cognitive impairment (MoCA < 12), the questionnaire responses for the persons were gathered by carer proxy. All persons with dementia attended the clinic with a carer and they were also present during the consultation. Carers were also asked select question items, such as whether continence care affects care they provide (and if so, to what degree), contact with community support, and any concerns regarding whether UI may be the principal reason for future NH admission.

**Figure 1:**
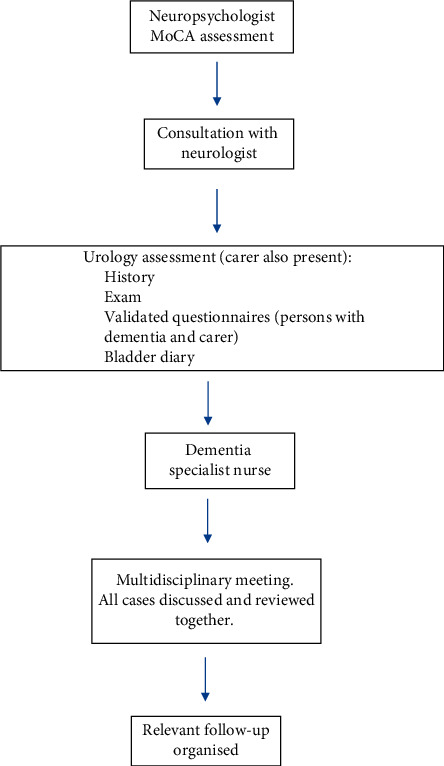
Flow diagram of the clinic set-up.

Referrals and liaison with bladder and bowel team were made if needed. All the cases were routinely discussed in a multidisciplinary team (MDT) format at the end of the clinic.  Figure 1 provides overview of the clinic pathway.

### 1.3. Evolution of Service and Challenges

In order to help recruitment and support identification of persons more suitable for assessment, electronic case notes were screened in advance, for example, if previous referrals from primary care or inpatient care summaries had mentioned urinary problems, but no formal evaluation had taken place. These were then highlighted to other team members as part of the clinic briefing. In addition, being asked by neurologists, persons were also asked at the end of Montreal Cognitive Assessment (MoCA) by the psychologist and given a large print card as a kind of “passport” reminder. A checklist sheet was later developed and held at reception to ensure the person was seen by the urology if required. The urologist later goes to spend time with the bladder and bowel team to learn their perspective on this issue as it emerged how valuable this resource was.

## 2. Results

The overall sample size was 150 (75 persons with dementia and 75 carers. Average age of persons with dementia was 72 years (range 56–98). The majority of persons with dementia (94.7%, *n* = 71) lived in the community with carer support.  Table 1 outlines baseline information. Tables 2 and 3 show responses to question items from persons with dementia and their carers, respectively. In addition to these results, 88% (*n* = 67) of persons with dementia felt the topic was socially embarrassing for them to discuss, both among their families, and with health professionals. Interestingly, those patients who responded that UI was not embarrassing had lived with the condition for a long time and stated that, in the beginning, it had indeed been very embarrassing. More than half of the patients (60%, *n* = 45) reported that they had not disclosed their struggles with incontinence previously. 34% had concurrent faecal incontinence.

Family caregivers were able to describe in great detail the first-time leakage had occurred in a public place. While all respondents stated that continence issues affected the care they provided; this was heightened in those who could not afford additional (self-funded) assistance compared to those who could (6.9/10 versus 8.3/10). Those from more socio-economically privileged backgrounds appeared to manage better as extra care and supplies could supplement standard resources.

## 3. Discussion

### 3.1. Key Findings

This coordinated approach to the management of dementia and bothersome urinary tract symptoms has revealed how much persons with dementia can struggle with UI. The disruption to daily life includes sleep, normal daily outings, and relationships with their community. This affects not only the person with dementia but also the care, which family and carers provide which was confirmed by everyone. Bladder and bowel continence teams appear to be an under-recognised resource, and therefore, it is key that awareness is raised accordingly.

### 3.2. How Do These Findings Compare with Relevant Literature?

Our findings were consistent with previous research by Engberg et al. with regard to the range of self-care behaviours persons with dementia adopt such as strict fluid restriction in an attempt to remedy UI [20]. The burden of UI appeared under-reported due to the common misconception that it is an inevitable part of the disease and ageing process and that no treatment strategies are available.

Samuelsson et al. reported how heavy a toll the financial elements of continence care can have [21]. We also found that worries regarding access to continence products and the associated financial burden weighed heavily in the minds of many family caregivers. One of the basic tests we performed included measurement of postvoid residual bladder volumes with a mobile scanner to rule out chronic urinary retention. While the latter is not generally clinically significant, sometimes it can be. Parson et al. evaluated feasibility of intermittent self-catheterisation (ISC) in elderly patients and reported the overall success rate was 82% in patients over 65 years of age [22]. In carefully selected cases, ISC can also be performed by a carer. While ISC may not be an option in patients with severe cognitive impairment, we found that patients with earlier/milder disease appear to still be suitable candidates. Research on continence in patients with cognitive impairment reveals that for disclosure of issues surrounding UI, the self-referral rate for UI among the elderly is lower [23]. Drennan et al. recorded that carers would try to preserve patient dignity and therefore tended to under-report the extent of the problem(s) [5]. We also found that time and active listening were required for patients to reveal continence problems. Cole et al. added to this by finding that patients often prefer not to complain as they do not want to burden the carer [23]. Of the many stigmas held by individuals and society, the shame associated with lack of self-control of urine and faeces can be very powerful [24], such as the human response to such a matter that powerful emotions and tensions can be triggered [25]. The impact of shame and embarrassment highlighted in these studies was consistent with our findings in this clinic.

### 3.3. Limitations and Future Research

Our study does not provide any estimate of prevalence of bothersome urinary symptoms among dementia patients. Questions asked during the assessment were drawn from validated questionnaires, which is a strength. However, in four persons with severe cognitive impairment (MoCA score <12), questions were answered by carer proxy. While this was only required in a few cases, this can introduce bias. This is a recognised difficulty in assessments related to quality of life in persons with dementia. Boyer et al. reported the potential discrepancies between persons and their carer proxy [26]. A novel solution for this to improve assessment of continence issues in persons with dementia is the “ICIQ Cog.” This is a patient-reported outcome measure (PROM) specifically designed for the assessment of urinary tract symptoms in patients with cognitive impairment, which is currently under development. While its properties have been finalised, it has yet to be formally validated for clinical use [13]. The final version will serve as useful outcome measure in future studies, particularly when assessing continence interventions. Given how valuable the use of a formal bladder diary is in the assessment of patients with bothersome urinary symptoms, development of a modified version for application in cognitively impaired patients, which can be completed by care giver, would be of benefit [19]. A validated pad diary would also be a valuable resource. While there is still value in using the current version available, our experience highlighted it is more suited to those with milder cognitive impairment.

Given the important role of community bladder and bowel services and the financial pressures faced by the National Health Service (NHS), it is imperative that such services are appreciated and supported through this.

This project, while limited by small numbers of persons with dementia, shows the potential benefits of a coordinated approach between specialities. In financially pressured healthcare systems, the next step in our hospital is for the development of a tailored referral pathway between neurologists and urologists to identify those persons with dementia who would benefit most from urology input. In addition to this, establishment of a referral pathway between neurology services and the bladder and bowel community services is also considered to be a valuable initiative to pursue.

## 4. Conclusion

In persons with dementia who develop UI, it can be distressing and disrupt many facets of daily life. Moreover, it can serve as the precursor to loss of independence and even early nursing home admission. Bladder and bowel services are invaluable and awareness of the benefits of such resources should be increased across primary and secondary care. Development and expansion of models like this one and improved interspeciality referral pathways are important steps improving care.

## Figures and Tables

**Table 1:** Summary of demographics and baseline information.

Overall sample size	150
Persons with dementia	75
Carer	75
Mean age	72 years (range 56–98)
Male-to-female ratio	1.6 : 1
Nursing home residency/community dwelling	4 (5.3%)/71 (94.7%)

*Dementia type*	
(i) Alzheimer's	57% (*n* = 43)
(ii) FTD	16% (*n* = 12)
(iii) Lewy body	10.7% (*n* = 8)
(iv) Vascular	9.3% (*n* = 7)
(v) Others, e.g., dementia associated with multiple sclerosis (MS) or progressive supranuclear palsy (PSP).	5.3% (*n* = 4)

Mean MoCA score	18/30 (range 9–25)
*Distibution of MoCA scores*	
Mild (20–24)	40% (*n* = 30)
Moderate (13–20)	55% (*n* = 41)
Severe (<12)	5%. (*n* = 4)

Concurrent faecal incontinence and urinary incontinence	34% (*n* = 18)

*Transient causes*	
Urinary tract infection	11% (*n* = 8)
Constipation	35% (*n* = 26)
Urinary retention	4% (*n* = 3)

Patients requiring changes to medication	39% (*n* = 29)
Referrals to bladder and bowel services	44% (*n* = 33)

**Table 2:** Responses of persons with dementia to question items.

Question	Response
How often do you leak urine?	
(i) About once a week	12% (*n* = 9)
(ii) Two or three times a week	21.3% (*n* = 16)
(iii) About once a day	18.7 (*n* = 14)
(iv) Several times a day	30.7% (*n* = 23)
(v) All the time	17.3% (*n* = 13)

How much urine do you leak?	
(i) Small amount	54.7% (*n* = 41)
(ii) Moderate amount	27% (*n* = 21)
(iii) Large amount	17.3% (*n* = 13)

How much does leaking of urine interferes with everyday life? (on a scale of 1–10)	Mean score 7.7/10 (range 2–10)

Does urine leakage interfere with your sleep?	
(i) Slightly	14.7% (*n* = 11)
(ii) Moderately	18.7% (*n* = 14)
(iii) A lot	25.3% (*n* = 19)
(iv) Not at all	31.3% (*n* = 31)

Are your daily activities limited due to incontinence?	
(i) Slightly	17.3% (*n* = 13)
(ii) Moderately	24% (*n* = 18)
(iii) A lot	41.3% (*n* = 31)
(iv) Not at all	17.3% (*n* = 13)

How much does your bladder problem make you feel more anxious?	
(i) Slightly	14.7% (*n* = 11)
(ii) Moderately	30.7% (*n* = 23)
(iii) A lot	21.3% (*n* = 16)
(iv) Not at all	33.3% (*n* = 25)

Is continence a socially embarrassing topic to discuss?	Yes = 88% (*n* = 67)

**Table 3:** Responses of carers to question items.

Question	Response
Does continence care affect the care you provide?	Yes = 100% (*n* = 75)
How much does incontinence affect the care you provide? (on a scale of 1 to 10)	7.7/10 (range 5–10)
Are you concerned that UI may be a principal reason for future nursing home admission?	Yes = 65% (*n* = 46)
Are you aware of or have you been in contact with community bladder and bowel services?	Yes = 30.7% (*n* = 23)
Would more awareness and teaching on continence care be beneficial?	Yes = 100% (*n* = 75)

## Data Availability

Data are available from the corresponding author upon request.
